# Optimized Semi-Interpenetrated p(HEMA)/PVP Hydrogels for Artistic Surface Cleaning

**DOI:** 10.3390/ma15196739

**Published:** 2022-09-28

**Authors:** Giulia Tamburini, Carmen Canevali, Silvia Ferrario, Alberto Bianchi, Antonio Sansonetti, Roberto Simonutti

**Affiliations:** 1 Department of Materials Science, University of Milano-Bicocca, 20125 Milan, Italy; 2 Institute for Heritage Science (ISPC), National Research Council (CNR), 20122 Milan, Italy; 3 Graftonica Srl, 20148 Milan, Italy

**Keywords:** p(HEMA), PVP, copper removal, maleic anhydride, copper chelator

## Abstract

The synthesis of hydrogels that are based on poly-hydroxyethyl methacrylate, p(HEMA), network semi-interpenetrated with linear polyvinylpyrrolidone (PVP) was optimized in order to allow both a fast preparation and a high cleaning effectiveness of artistic surfaces. For this purpose, the synthesis parameters of the gel with PVP having a high molecular weight (1300 kDa) that were reported in the literature, were modified in terms of temperature, time, and crosslinker amount. In addition, the gel composition was modified by using PVP with different molecular weights, by changing the initiator and by adding maleic anhydride. The modified gels were characterized in terms of equilibrium water content (EWC), water uptake, conversion grade, and thermal properties by differential scanning calorimetry (DSC). The cleaning effectiveness of the gels was studied through the removal of copper salts from laboratory-stained specimens. Cleaning materials were characterized by electron paramagnetic resonance (EPR) spectroscopy, ultraviolet-visible (UV-Vis) spectroscopy, and inductively-coupled plasma-mass spectrometry (ICP-MS). Cleaning was assessed on marble specimens by color variation measurements. The gel synthesis is accelerated by using PVP 360 kDa. The addition of maleic anhydride in the p(HEMA)/PVP network allows the most effective removal of copper salt deposits from marble since it acts as a chelator towards copper ions.

## 1. Introduction

Gels are materials that are composed of long interconnected chains that trap a fluid; therefore, they are used for many applications, including fillers for prostheses [1], and soft contact lenses [2]. Recently, gels have gained an important role also for the cleaning of art surfaces, mainly for their ability in controlling and confining solvent release, besides providing a physical removal of solid micro-fragments [3]. Currently, agar hydrogels are among the most used materials for the cleaning of cultural heritage surfaces. They are physical gels with weak bonds among the polymeric chains, thus they can respond to heat treatments or be disrupted by mechanical forces [4]. They are very versatile in removing different types of soiling from various substrates, have a low impact on the artworks, and a low cost [5,6].

The cleaning issue of removing copper stains from artwork surfaces with agar gels has been recently studied on marble laboratory specimens that were stained with brochantite in well-controlled and reproducible conditions [5,7,8,9,10,11]. Brochantite, CuSO_4_ 3 Cu(OH)_2_, is one of the most frequently occurring compounds in outdoor bronze corrosion patinas. Marble laboratory-stained specimens proved to be the best surfaces for modelling a copper-stained heritage surface. In fact, copper compounds are equally distributed on them, with good adhesion to the substrate [12], thus allowing a systematic comparison of the tested gels in terms of cleaning effectiveness. It was shown that copper coordination is the cleaning driving force, and that stain removal was about twice as effective by using agar gels containing chelating agents, such as ethylene-diaminetetraacetic acid (EDTA) or ammonium citrate tribasic (TAC), than using pure gels [10,12]. Due to the possibility of water confinement, agar gels were successfully also used for removing generic soiling from canvases, stains from paper, and soluble salts from mural paintings and plaster [8,12,13,14]. 

However, better water confinement can be achieved by using chemical gels, which have a three-dimensional network that is characterized by covalent bonds [3]. The main feature of chemical gels is high retentivity, which deals with the polymer matrix’s ability to release fluids in a controlled manner, avoiding the uncontrolled diffusion of solvents to the artefact. Moreover, chemical gels exhibit excellent mechanical properties, thus they can be completely removed from the artistic surface after the cleaning treatment, without leaving any residues. Acrylamide hydrogels were successfully applied for the cleaning of easel paintings [15]. 

Semi-interpenetrating (semi-IPN) polymers are even more complex cleaning materials, based on polymer blends in which linear or branched polymers are embedded into one or more polymer networks, without any chemical bonds between them [16]. An important feature of semi-IPN hydrogels is the possibility of tuning their composition (monomer, cross-linker, liquid medium, etc.) and the quantitative proportions of their constituents (e.g., cross-linker, water, etc.) to obtain gels with tailored properties [17]. Innovative semi-IPN hydrogels that are based on linear polyvinylpyrrolidone (PVP) embedded into poly (2-hydroxyethyl methacrylate), p(HEMA), possess suitable water release and retention properties. Moreover, they are biocompatible, with low environmental toxicity, and are transparent and easy to manipulate [18,19]. Due to their properties, these hydrogels are used for biomedical purposes, including controlled drug release [20,21,22,23,24]. Various types of p(HEMA)/PVP semi-IPN hydrogels with PVP average Mw 1300 kDa and different component ratios (water, PVP, HEMA, and crosslinker amounts) were studied in the literature for a controlled and efficient cleaning of art surfaces, and it was observed that the “H65” gel, containing 65% (*w*/*w*) water, possesses the best swelling capacity in water [25]. However, the synthesis procedure of the H65 gel is not fast, since it takes 8 h for PVP 1300 kDa to dissolve in water, and its effectiveness in artefact surface cleaning suffers from an excessive wetting action [25]. 

**Figure 1:**
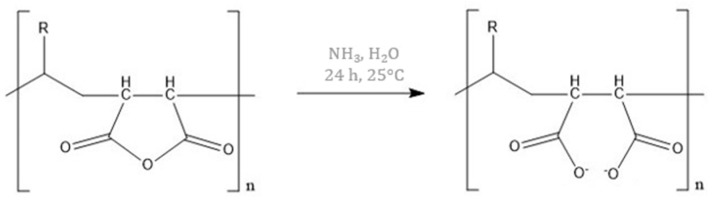
Ring-opening mechanism of the maleic anhydride inside the p(HEMA) network in a basic environment.

Thus, the objective of the present article is to optimize the H65 gel synthesis, in order to: i) allow a faster preparation, and ii) improve the effectiveness in the cleaning of art surfaces. These aims were obtained by varying the synthesis parameters (temperature, time, and crosslinker amount) and the chemical composition (PVP molecular weight, type of intercalated polymer and of initiator). As for the variation in the chemical composition, it is important to emphasize the novelty of the addition of maleic anhydride (MA, Figure 1) within the network, which remains anchored to the gel framework without leaving residues [26], this issue being very important both for the artwork and for the environment. Maleic anhydride has never been used before as an additive in the field of cultural heritage, but it has only been used in the production of resins or in the field of pharmaceuticals [27], coatings, and as an additive in plastics [28].

Moreover, MA can act as a chelator for metal ions: in the presence of a basic environment (such as an aqueous solution containing NaOH or NH_3_), MA opens the ring forming a salt (Figure 1), and in this conformation, it is able to form complexes with metal centres [29]. Thus, its addition in H65 gels could improve the surface cleaning effectiveness by acting as a chelator of metal ions that are deposited on the artefacts.

Gels were characterized from a chemical-physical point of view by differential scanning calorimetry (DSC). Then, the cleaning effectiveness was studied after contact with marble laboratory specimens that were stained with brochantite. The coordination of Cu(II) centres within the different gels was studied by electron paramagnetic resonance (EPR) spectroscopy. Then, the copper removal was quantified on the solutions that were released from the gels by ultraviolet-visible (UV-Vis) spectroscopy and by inductively-coupled plasma-mass spectrometry (ICP-MS). Finally, the effectiveness of cleaning was studied on marble laboratory specimens through color variation measurements.

## 2. Materials and Methods

### 2.1. Materials

Poly(vinylpyrrolidone) (PVP) with an average Mw 1300 kDa (>99%) was obtained from Alfa Aesar (Ward Hill, MA, USA). PVP with Mw 360 kDa and Mw 40 kDa, 2-hydroxyethyl methacrylate (HEMA) (>97%), α,α′-Azoisobutyronitrile (AIBN), and N,N-methylene-bis(acrylamide) (MBA) (>99%) were purchased from Sigma-Aldrich (St. Louis, MI, USA). HEMA was purified by filtration through an activated basic alumina column, while AIBN was purified by recrystallization from methanol prior to use. Maleic anhydride (MA) (>99%) was obtained from Fluka (New Rochelle, New York, NY, USA). For pH solution modification, ammonium hydroxide solution 33% Riedel-de Haën, (New Rochelle, New York, NY, USA) was used. HNO_3_ (70%, Carlo Erba, Cornaredo, Italy) was employed for releasing solutions from gels. MQ water^®^ and ultra-pure water were used when necessary. 

### 2.2. Gel Synthesis

Unless otherwise specified, the semi-IPN p(HEMA)/PVP gels were synthesized using aqueous solutions containing HEMA monomer, MBA, and PVP as semi-interpenetrating polymer; AIBN was used as a radical initiator.

All the products were mixed in the proper amounts (Table 1) and the solutions were bubbled with nitrogen for 20 min to remove dissolved oxygen, which could inhibit the radical polymerization of HEMA. Then, the solutions were sonicated for 30 min to eliminate the remaining gas bubbles and poured into Petri dishes having 0.5 cm thickness and 11.0 cm diameter. The polymerization reaction occurred at 60 °C for 4 h, after which a transparent and rigid gel was obtained. After polymerization, hydrogels were washed with water, then placed in containers that were filled with distilled water. The water was renewed twice a day for 5 days to remove any residue of both unreacted HEMA and free PVP.

For what concerns HMA gel, it was immersed in an aqueous solution containing ammonia (28%) to reach a pH 10.0 value prior to use it as a cleaning agent, in order to induce the opening of the maleic anhydride ring. 

### 2.3. Preparation and Cleaning of Laboratory Specimens

Specimens that were sized 4.5 cm × 5.0 cm × 2.0 cm of white Carrara marble were used. Each specimen’s most homogeneous surface (4.5 cm × 5.0 cm) was selected and processed as previously described in the literature [4]. This surface was stained by in situ syntheses of brochantite, Cu_4_(SO_4_)(OH)_6_. For this purpose, marble specimens were immersed in 0.05 M (1.2% *w*/*w*) solutions of CuSO_4_ 5H_2_O in water at 55 °C, and then an equimolar solution (0.53% *w*/*w*) of Na_2_CO_3_ was added dropwise. After the addition was complete, the marble specimens remained immersed overnight in the reaction solution and were successively allowed to dry in the air. For cleaning operations, gels that were sized 2.0 cm × 5.0 cm × 0.5 cm were cut, applied to the stained surfaces displayed horizontally, and left for 60 min. This procedure allowed treatment under well-controlled and reproducible conditions.

### 2.4. Gel Characterization

The degree of conversion was calculated as the ratio between the quantity of reacted HEMA (obtained by heating gels in an oven at 100 °C to eliminate water and by subsequently measuring their mass) and the initial HEMA quantity.

Swelling measurements were performed by placing the gel in Petri dishes with distilled water for 7 days, and by measuring the mass values every 24 h.

The equilibrium water content (EWC) is the amount of water that is retained within the gel. It can be determined by Equation (1):

(1) $$\mathrm{EWC}\;\left(\%\right)=\frac{W_{W}-W_{0}}{W_{W}}\times 100\%$$

where W_0_ is the weight of the gel immediately after synthesis and W_w_ is the weight of the water-swollen gel in equilibrium that was obtained at least 7 days after the polymerization reaction [30].

The water uptake, EWC and degree of conversion values are affected by ± 1% error. DSC analysis of gels was performed with a Mettler Toledo Star (Milan, Italy) thermal analysis system that was equipped with a liquid N_2_ low-temperature apparatus. All the samples were first dried in an oven overnight at 80 °C to remove water, then they were weighed, and a fixed amount (about 5.50 ± 0.05 mg) was sealed into 40 μL aluminium pans. Heat flow was measured during sample heating from −120 °C to 200 °C at 20 °C/min under N_2_ atmosphere. Indium was used as a standard for temperature and heat flow calibration. Glass transition temperature (T_g_ ± 1 °C) was obtained as the midpoint of the step transition in the calorimetric curve.

The electron paramagnetic resonance (EPR) spectra of the gels were recorded at −150 °C at the X-band frequency on a Bruker EMX EPR spectrometer that was equipped with a BVT 2000 variable temperature unit (Bruker, Germany). The gels were inserted into quartz tubes having an internal diameter of 3 mm. Since it was not possible to compact gels at the bottom of the EPR tubes, any quantitative comparison among different samples was avoided. Unless otherwise indicated, the modulation frequency of 100 kHz, modulation amplitude of 1 G, and microwave power of 5 mW were used. The g values were determined by standardization with α,α’-diphenyl-β-picryl hydrazyl (DPPH) radical. 

The UV-Vis absorbance spectra were recorded at room temperature on an Agilent (USA) Cary 100 spectrophotometer in the spectral range from 190 to 900 nm. Spectra were recorded on solutions in quartz cuvettes with 1 cm optical paths. There were two kinds of samples that were investigated: (i) solutions that were released from gels and (ii) “blank” solutions. The released solutions were obtained in the following way: gels were weighed, immersed in a constant volume (50 mL) of an acidic solution (H_2_O:HCl 1:1 *v*/*v*) for 12 h, and then the released solutions were recovered and characterized. For what concerns the “blank” samples, acidic solutions (H_2_O:HCl 1:1 *v*/*v*) were prepared containing the following: (i) CuSO_4_; (ii) HEMA, PVP, and CuSO_4_; and (iii) HEMA and PVP. All solutions had the same volume (50 mL) and the same concentration of CuSO_4_, PVP and HEMA was fixed at 10^−3^ M in order to obtain intense absorbance values.

The solutions that were released from gels were also analyzed by an inductively-coupled plasma-mass spectrometer (ICP-MS, ThermoFisher iCAP Q, Waltham, MA, USA). Ultra-pure water that was produced by a Sartorius arium mini system and ultrapure nitric acid, produced via sub-boiling distillation with DuoPUR-Milestone equipment [31], were used to dilute the samples and prepare the standard solutions. Procedural blanks and control standards were also analyzed during each analysis batch. The data (± 2% error) were normalized according to each sample weight.

Photographic images were taken with a Nikon D3300 camera that was equipped with AF-P NIKKOR 18–55 mm f/3.5–5.6 G lens.

### 2.5. Marble Specimen Characterization 

Color measurement data were acquired using a Konica Minolta Chromameter CM−700 d with D65 source and d/8 analytic geometry in the CIE L*a*b* system, measuring a circular area corresponding to a 6 mm diameter. The parameter L* represents the lightness from 0 (black) to 100 (white); a* denotes the red/green values and b* the yellow/blue values, both ranging from +60 to −60. For each specimen, 25 measures were acquired. The total color difference can be stated as a single value, expressed as in Equation (2):

(2) $${\Delta E}^{*}=\sqrt{{\left({\Delta L}^{*}\right)}^{2}+{\left({\Delta a}^{*}\right)}^{2}+{\left({\Delta b}^{*}\right)}^{2}}$$

where ΔL*, Δa*, and Δb* are the differences between the values that were obtained after and before contact with gels for L*, a*, and b*, respectively.

The ΔE* value represents the distance of two color points on the CIE L*a*b* color space [32,33].

## 3. Results

### 3.1. Optimization of Gel Synthesis

The H65 is defined as a semi-IPN hydrogel according to the literature [25]; in fact, it is constituted of an ‘‘alloy” of cross-linked and linear polymers. The cross-linked network is constituted by p(HEMA), which is synthesized in the presence of linear PVP chains. As it is known, the chemical process of the synthesis provides that HEMA and MBA monomers, having double bonds, react very fast in the presence of the radical initiators that are generated by the homolysis of AIBN. Moreover, MBA monomers, participating in the free radical polymerization with two double bonds, act as crosslinkers. On the other hand, PVP chains, not having double bonds available, should display a very limited reactivity during the polymerization, in agreement with the literature [25], which reported that no chemical reactions occur between linear and branched polymers in semi-IPNs. On the other hand, Melnyk et al. reported on the synthesis of PVP-graft-poly(HEMA) hydrogel membranes in experimental conditions that were similar to those that were used in the present paper [24]. Indeed, the existence of a limited number of grafting points on PVP chains do not significantly alter the structure of semi-IPN hydrogels, since the PVP segments starting from the grafting point are long enough to behave as free polymer chains. Such PVP segments cannot be removed by extraction from the gel, particularly in the case of very high molecular weight PVP, as the ones that were used in the present paper.

The H65 gel was synthesized according to the composition that was reported in the literature [25]: PVP 1300 kDa (24.5%), water (64.9%), HEMA (10.5%), MBA as a crosslinker (0.21%), and AIBN as an initiator with a monomer/initiator ratio of 1:1 × 10^−2^. It took 8 h for PVP 1300 kDa to dissolve in water, then polymerization occurred at 60 °C for 4 h.

In order to optimize the gel synthesis, an attempt was made to modify the H65 parameters and reagents that were reported in the literature (Table 1). In particular, the synthesis parameters, the composition of the network, and the polymeric species that intercalate in the network were modified.

In order to improve the gel characteristics, the synthesis parameters (temperature, time, and crosslinker amount) were changed. The temperature of hydrogel synthesis should remain below 70 °C in order to avoid undesirable exothermic effects, in agreement with the literature [24]. In fact, since polymerization takes place in water, above 70 °C a significant evaporation occurs, and polymerization could be driven only in an autoclave. Thus, we decided to maintain the same experimental set-up for polymerization, testing the two temperature values of 60 °C (literature value) and 70 °C (gel H70C).

For what concerns the polymerization time, we decided to test 16 h (gel H16h), which represents a very large increase with respect to the time that was reported in the literature (4 h), in order to assess any change in extreme time conditions.

Finally, the amount of cross-linker was increased twice, three times and four times (gels HMBA2, HMBA3, and HMBA5, respectively), according to a consolidated procedure in polymer synthesis.

Instead, the changes to the composition were proposed to understand whether it was possible to replace PVP 1300 kDa with other polymeric species that were more soluble in water and thus with a faster dissolution. Among the several commercial PVP, we chose to compare PVP with a very high molar mass (1300 kDa) to PVP with a very low molar mass (40 kDa, giving gel H40) and an intermediate value (360 kDa, giving gel H360). For all these hydrogels, the synthesis procedure is identical to that which was reported above for H65 gel, except that PVP 1300 was fully replaced with the different polymer species that were mentioned before. The possibility of modifying the composition of the p(HEMA) network, by using a water-soluble initiator (4,4’-azobis (4-cyanovaleric acid)) (ACVA) (gel HACVA), or by adding maleic anhydride (MA) (gel HMA) was also investigated. In the former, the monomer/initiator ratio was maintained equal to the one that was reported in the literature [25], while for the latter the maleic anhydride was added in a 9:1 ratio with the HEMA monomer.

### 3.2. Gel Characterization

The optimization of the gel synthesis was assessed by relating the changes that were described in Section 3.1 to EWC, water uptake, and conversion degree (Table 2).

As it is known, EWC provides important information on the state of the gel and is useful for verifying its performance during the swelling—dehydration—swelling cycle, which reproduces the procedure for applying the gel itself; it can also provide information on pore size and its evolution during swelling and drying. In fact, porosity is one of the main characteristics of gels because it can affect other important properties such as the retentive power of the solvent, the optical properties (transparency, opacity, translucency), and the ability to recall back the dissolved soiling substances through capillary suction.

**Figure 2:**
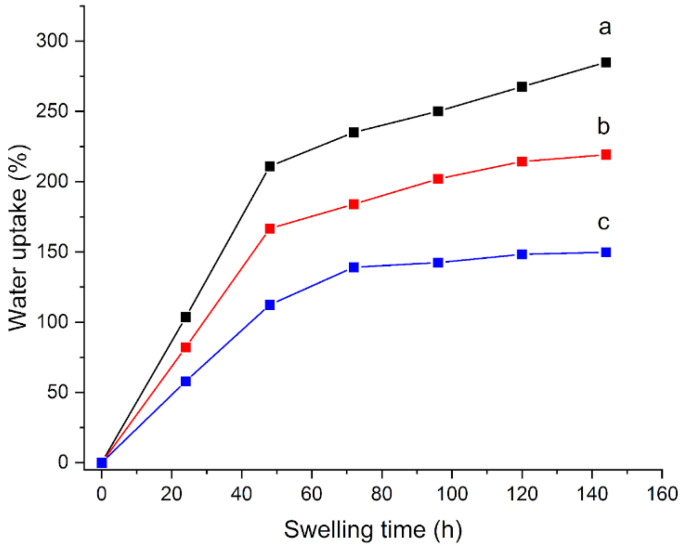
Water uptake (%) as a function of swelling time for (**a**) H65, (**b**) H70C, and (**c**) H16h gels that were immersed in water.

As a first modification, the temperature of polymerization from 60 to 70 °C (H70C) was increased. The swelling curves that were obtained for these hydrogels are shown in Figure 2: after 24 h, the H65 gel shows a water uptake of 103% (Figure 2a), while H70C shows a smaller water uptake of 82% (Figure 2b); correspondingly, an increase in the degree of conversion from 72 to 94% is observed (Table 2).

On the other hand, the increase in the polymerization time from 4 to 16 h (H16h) causes a decrease in the degree of conversion to 60%, and an ability to absorb less water, with a water uptake of 58% after 24 h (Figure 2c). The EWC that was calculated from Equation (1) resulted in 167% for H70C and 152% for H16h, smaller values compared to 215% of H65 (Table 2).

Thus, the tested changes in temperature and time of polymerization did not give any improvement with respect to the standard EWC, water uptake, and conversion degree values [25], thus these parameters remained unchanged in the subsequently prepared gels.

**Figure 3:**
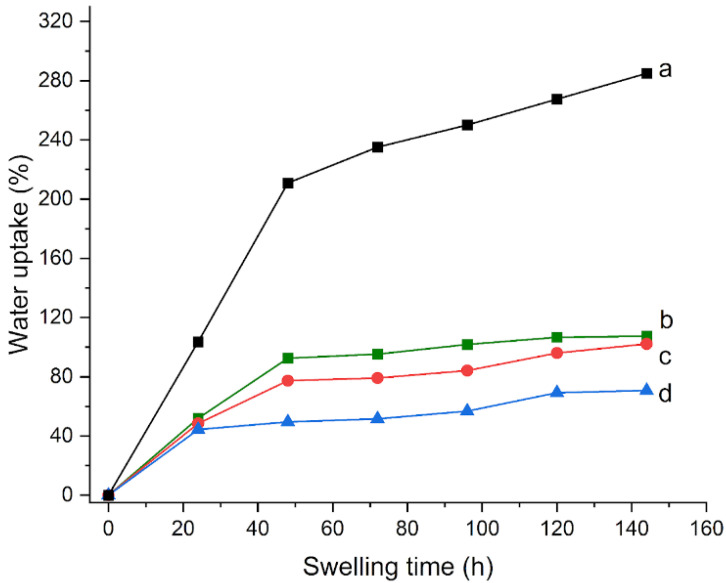
Water uptake (%) as a function of swelling time for (**a**) H65, (**b**) HMBA2, (**c**) HMBA3, and (**d**) HMBA5 gels that were immersed in water.

Then, the amount of crosslinker (MBA) was increased with respect to the original composition: HMBA2, HMBA3, and HMBA5 gels were synthesized, in which the amount of MBA is increased twice, three, and five times, respectively. The reference curve for H65 is reported in Figure 3a. As the MBA content increased, a decrease in both the water uptake, from 51 to 44% (Figure 3), and conversion degree values, from 53 to 48% (Table 2), were observed. This result could be explained by considering that the higher crosslinking limits the volume variation ability during swelling in solution. In addition, gels become visually more opaque as the amount of crosslinker increases; this can be explained by assuming that, by increasing the initiator, numerous shorter chains can be formed that make the gel more viscous. Therefore, a gradual decrease of EWC from 92 to 70 to 49% is calculated as the amount of crosslinker was doubled, tripled, and quintupled, respectively (Table 2).

Since it was verified that the increase in the crosslinker amount did not give any improvement to the gel synthesis with respect to the standard value [25], this parameter remained unchanged in the subsequently prepared gels.

**Figure 4:**
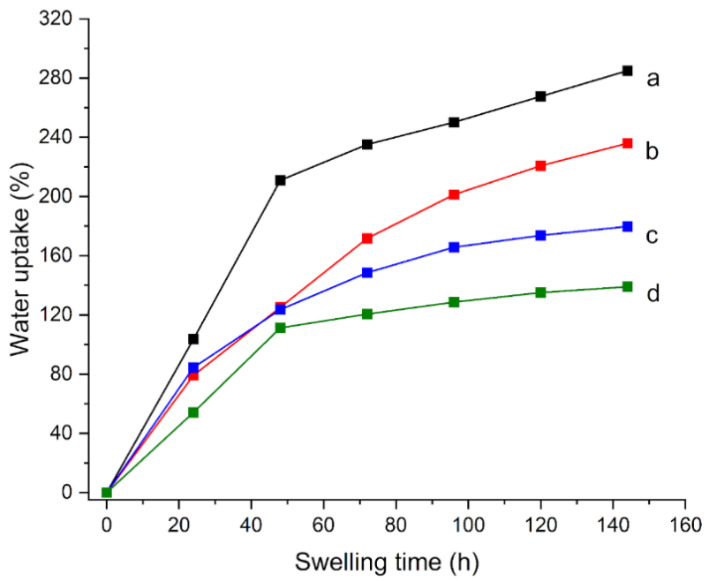
Water uptake (%) as a function of swelling time for (**a**) H65, (**b**) H360, (**c**) HMA, and (**d**) HACVA, all immersed in water.

The polymers intercalated in the p(HEMA) network were also modified. As a substitution of PVP 1300, two different PVPs having 360 and 40 kDa molecular weights were used, obtaining gels H360 and H40, respectively. It was observed that the addition of PVP 360 (H360) allows a faster dissolution in water with half dissolution times with respect to PVP 1300 (4 h and 8 h, respectively), with good results: the water uptake and the degree of polymerization resulted in 79% after 24 h (Figure 4b) and 86% (Table 2), respectively. The EWC for this hydrogel was 200%, a slightly lower value than that which was calculated for H65 (215%). These results showed that H360 gel deserves a deeper characterization.

On the contrary, for the H40 gel, it was not possible to carry out any characterization, since the intercalated polymer is so short that it cannot penetrate the p(HEMA) network, and, therefore, the final gel is non-rigid and unusable for the present scope.

The addition of maleic anhydride in a 9:1 ratio with HEMA (gel HMA), and the replacement of AIBN with ACVA (4,4’-azobis (4-cyanovaleric acid)) in gel HACVA resulted in the water uptake curves that are reported in Figure 4c,d, respectively. The results were positive for HMA: the water uptake and degree of conversion were 85% and 84%, respectively. The EWC value that was calculated for this hydrogel was 170%. Instead, it was decided not to consider HACVA for further characterization, despite a high degree of conversion (96%) and a high EWC (160%), since its water uptake is too low (25%) for an effective application to the cleaning of artistic surfaces.

### 3.3. Thermal Behavior of Optimized Gels

The optimization of H65 synthesis allowed us to identify H360 and HMA as the most promising gels, deserving better characterization. Thus, the thermal behaviour of these gels was studied through DSC measurements and compared to that of gel H65 (used as reference) and of the constituent polymeric species, p(HEMA) and PVP. The obtained thermograms showed high glass transition temperatures for all the gels, from 124 °C of p(HEMA) to 181 °C of PVP 1300 kDa (Table 3). It can be observed that the T_g_ values of all the gels (H65, H360, HMA) are intermediate between those of the constituent polymeric species (p(HEMA) and PVP). This characteristic derives from the method of gel preparation: having been synthesized by semi-interpenetration, they have final properties that correspond to the average of those of the single homopolymers. Moreover, it is possible to observe that the addition of maleic anhydride as a comonomer inside the p(HEMA) does not modify the thermal properties of the gel, since the T_g_ of the HMA gel (167 °C) is comparable to the one that was observed for the H65 and H360 gels (168 and 169 °C, respectively).

### 3.4. Study of the Cleaning Effectiveness

According to previous investigations, the following gels were selected for testing the cleaning effectiveness: H65 (used as reference), H360 and HMA. These gels were contacted with Carrara marble laboratory-stained specimens with brochantite for 60 min, then gels were removed, and characterization was performed on both gels and marble specimens. Gels were studied by EPR spectroscopy, while solutions released from gels were characterized by UV-Vis spectroscopy and ICP-MS spectrometry. Marble specimens were characterized by color measurements.

#### 3.4.1. Gel Characterization

**Figure 5:**
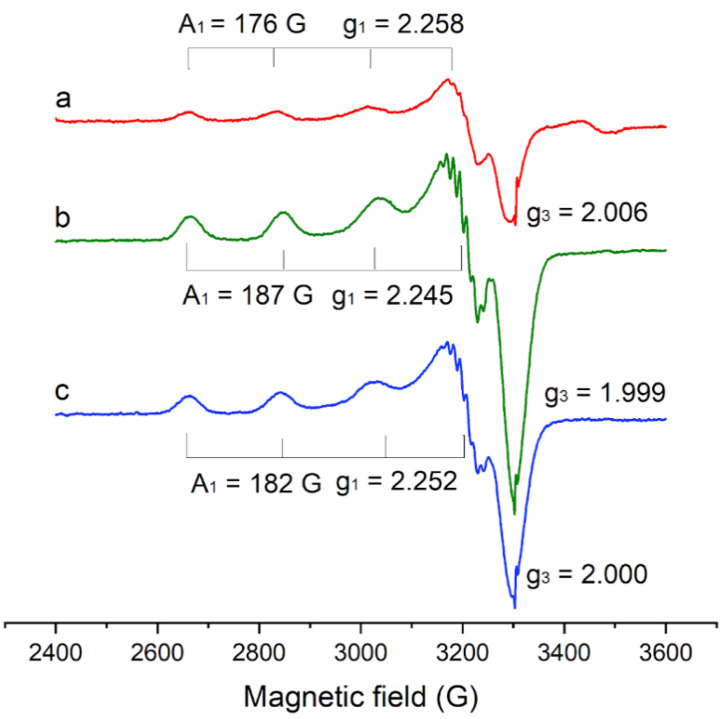
EPR spectra of gels after 60 min contact with laboratory-stained marble specimens: (**a**) H65, (**b**) H360, and (**c**) HMA.

In order to compare the coordination mode of Cu(II) centres within the different gels, EPR characterization was performed. Before contact with marble specimens, no EPR signal was detected. The spectra that were obtained after contact with stained marble specimens are reported in Figure 5 and their spectral parameters are reported in Table 4. Since it was not possible to compact gels at the bottom of the EPR tubes, any quantitative comparison among different samples was not feasible.

All the gels (H65, H360, HMA) displayed orthorhombic resonances, consistent with those of magnetically-diluted Cu(II) centres in a distorted tetragonal symmetry field of oxygen atoms [33]. This result shows that all the studied gels coordinate Cu(II) centres. It was reported that agar gels also coordinate copper centres [5,7,10,11,12].

The spectral parameters of the Cu(II) centres in the three gels are not significantly different. However, small, interesting variations can be observed: the value of g_1_ gradually decreases from 2.258 (H65) to 2.252 (HMA) to 2.245 (H360), while the value of A_1_ correspondingly increases from 176 G to 182 G to 187 G. In general, the decrease in g_1_ values that was accompanied by the increase in A_1_ value is related to an increase in tetragonal distortion of copper centres [34,35]. Thus, it could be argued that the tetragonal distortion of the metal centres very slightly increases from H65 to HMA to H360, and that distortion in HMA lies between that in H65 and H360 gels, confirming that the addition of MA does not modify the general characteristics of gels, or for the coordination mode of Cu(II) centres.

**Figure 6:**
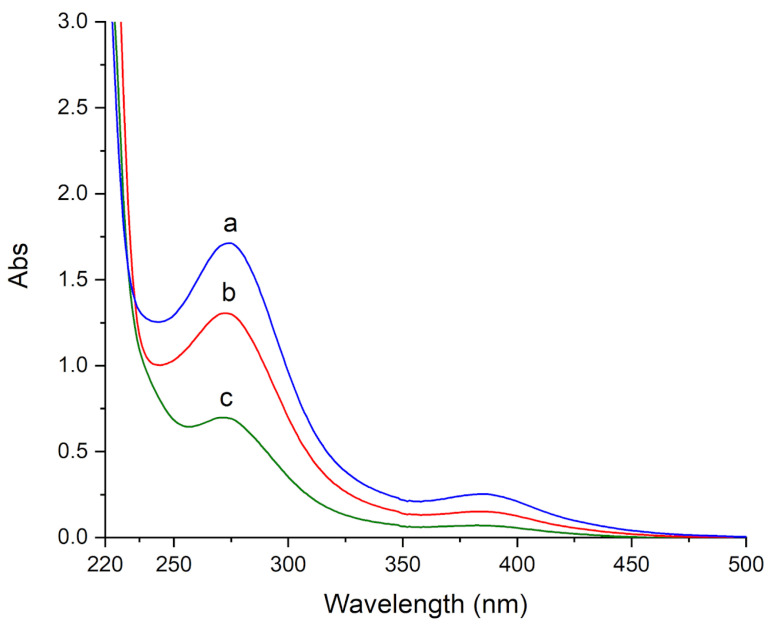
UV-Vis absorbance spectra of the released acidic solutions from (**a**) HMA, (**b**) H360, and (**c**) H65 gels.

In order to have a relative estimation of the cleaning effectiveness, the three investigated gels were contacted with marble specimens, removed, and soaked in an aqueous acidic solution (H_2_O/HCl 1:1 *v*/*v*) for 12 h. Then, the released solutions were recovered and UV-Vis absorbance spectra were recorded. The spectra showed very strong bands below 500 nm. For all gels, two peaks at 275 and 386 nm were observed, which on the basis of their position can be attributed to charge-transfer bands of copper complexes (Figure 6). In addition, a very weak and broad band at 800 nm, attributable to the d-d transition of Cu(II), and a very intense band at 218 nm, attributed to residual PVP that was released by the gels, were also observed, however are not shown in the figure for sake of clarity. 

**Figure 7:**
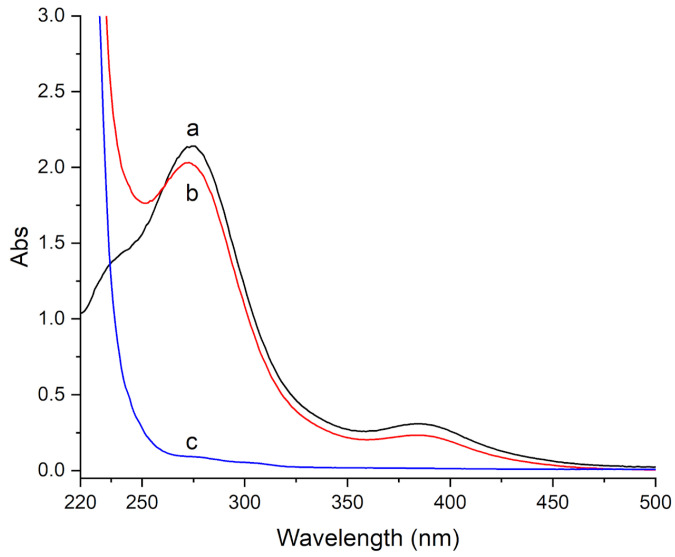
UV-Vis absorbance spectra of acidic solutions containing (**a**) CuSO4; (**b**) HEMA, PVP, and CuSO4; and (**c**) HEMA and PVP.

The spectrum of the solution that was released from the HMA gel shows the most intense bands at 275 and 386 nm (Figure 6a), followed by H360 and by H65 gels (Figure 6b,c, respectively). The same bands at 275 and 386 nm were also observed for an aqueous acidic solution (H_2_O/HCl 1:1 *v*/*v*) containing copper sulphate, together with a “shoulder” at 250 nm (Figure 7a). Thus, the bands at 275 and 386 nm can be attributed to Cu(II) chloride complexes [36]. It is known that several copper chlorides can form ([CuCl]^+^, [CuCl_2_]^0^, [CuCl_3_]^−^, [CuCl_4_]^2−^, and [CuCl_5_]^3−^), increasingly important as the chloride concentration rises [36]; however, the precise speciation is difficult to achieve and out of the scope of the present paper.

As expected, the 275 and 386 nm bands of copper chloride complexes were also observed for acidic solutions containing HEMA and PVP in the presence of copper sulphate (Figure 7b), being instead not detected in the absence of copper sulphate (Figure 7c). 

These results suggest that the HMA gel allows the most effective cleaning of brochantite-stained specimens, followed by H360 and by H65.

**Figure 8:**
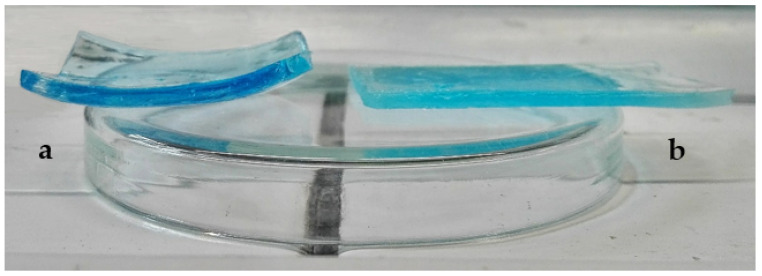
Photographic images of HMA (**a**) and H65 (**b**) gels after contact with marble specimens for 60 min.

To quantify the absolute copper concentration that was removed by the HMA gel, the acidic solutions that were released from gels were analyzed by ICP-MS. Before contact with marble specimens, only negligible copper amounts were detected. After 60 min contact with marble specimens, the amount of copper that was detected in solution from the HMA gel was 29 μg/cm^2^ of gel surface area, four times higher than the solution from H65 (7.2 μg/cm^2^). The HMA gel also showed a more intense color in the blue palette (Figure 8a) than the H65 gel (Figure 8b), confirming the removal of a greater amount of copper. These results show that MA in the HEMA network greatly improves the effectiveness in the removal of copper salt deposits from marble, probably because it acts as a chelator towards metal centres.

#### 3.4.2. Marble Specimen Characterization

Marble specimens were characterized both before (t_1_) and after (t_2_) contact for 60 min with HMA and H65 gels. The color measurement values are reported in Table 5. The total color variation (ΔE*) is very similar after contact with the two gels: 6.15 and 5.74 for H65 and HMA, respectively. However, some interesting considerations can be made by considering the single color parameters. In fact, ΔL* is quite different on specimens after contact with the two tested gels (4.8 and 0.93 for H65 and HMA, respectively), and the specimen that was contacted with H65 gel displays a greater value, bringing back the marble surface to a lighter color condition (closer to white), probably because the initial point was darker. The Δa* result (red-green axis) should be stressed: it resulted 3.82 and 5.66 for H65 and HMA, respectively. This result shows that the color variation that was obtained by the HMA gel is greater, informing of a more effective removal of the green components and confirming that the HMA gel displays a higher effectiveness for the removal of copper stains.

Negligible differences were detected for the Δb* parameter (0.22 and −0.32 for H65 and HMA, respectively), which measures the differences on the yellow-blue axis.

**Figure 9:**
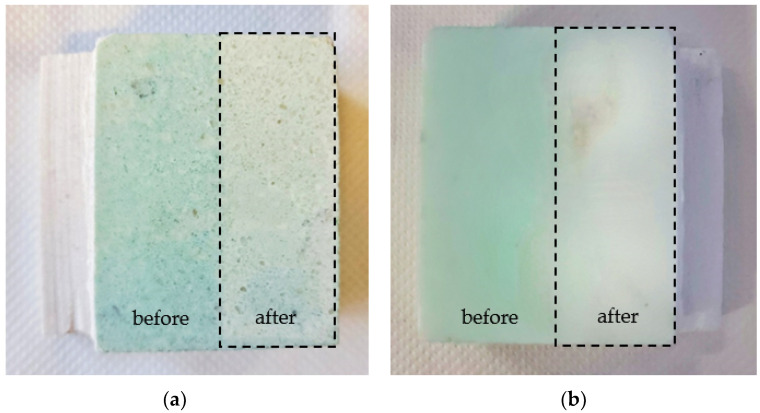
Photographic images of marble specimens before and after contact for 60 min with H65 (**a**) and HMA (**b**) gels. Dotted squares enclose the cleaned areas.

Photographic images confirm the color measurement results, showing for HMA a higher effectiveness for the removal of copper stains (Figure 9). Moreover, no gel residues were observed on surfaces after contact with gels.

## 4. Conclusions

The p(HEMA)/PVP semi-interpenetrating hydrogels are very useful tools for cleaning artistic surfaces since they are biocompatible, with low environmental toxicity, and are transparent and easy to manipulate. Moreover, they display a very high retentiveness, and their properties can be tailored through an appropriate modification of their synthesis. Unfortunately, the synthesis procedure of the H65 p(HEMA)/PVP hydrogel, reported in the literature, is not fast, and the effectiveness in artistic surface cleaning suffers from an excessive wetting action. The changes that were described here to the original synthesis allowed a faster preparation and an increase in the effectiveness of cleaning art surfaces, while maintaining high values of EWC, water uptake and conversion degree, and not modifying the thermal properties of the gels. 

In particular, the use of PVP with a molecular weight of 360 kDa (H360) accelerates synthesis operations, with half dissolution times with respect to the original H65 gel with PVP 1300 kDa (4 h and 8 h, respectively). Such a hydrogel allows an improvement in the effectiveness in cleaning artistic surfaces with respect to the original gel, as assessed on marble specimens that were stained with brochantite, which simulates the condition of stones in contact with bronze artefacts. The most effective copper removal from marble surfaces was obtained with the addition of maleic anhydride (MA) as a comonomer inside the p(HEMA)/PVP network (HMA). In particular, HMA displays a copper removal that is four times higher than the original gel H65, probably because MA acts as a chelator towards metal centres. This result is important, since it is known that metal coordination is the driving force for metal stain removal and MA was used here for the first time in a cleaning formulation in the cultural heritage field. 

It can, therefore, be concluded that the present paper shows the ways to fasten the synthesis of p(HEMA)/PVP hydrogels and to improve their cleaning action on artistic surfaces. Further investigation can provide a deeper understanding of its applicability for other applications, including the cleaning of other surfaces (i.e., water-sensitive artefacts such as paper, canvas, etc.), and the removal of other metal compounds (i.e., of iron, calcium, etc.).

## Figures and Tables

**Table 1:** Summary of the synthesized gels with modifications to the original H65 formulation.

Gel	Polymerization Temperature (°C)	Polymerization Time (h)	PVP Average M_w_ (kDa)	MBA (%)	Type of Radical Initiator	MA/HEMA	Modifications with Respect to H65
H65 [25]	60	4	1300	0.21	AIBN	0	−
H70C	70	4	1300	0.21	AIBN	0	Polymerization temperature 70 °C
H16h	60	16	1300	0.21	AIBN	0	Polymerization time 16 h
HMBA2	60	4	1300	0.42	AIBN	0	Twice the MBA content
HMBA3	60	4	1300	0.63	AIBN	0	Three times the MBA content
HMBA5	60	4	1300	1.05	AIBN	0	Five times the MBA content
H360	60	4	360	0.21	AIBN	0	PVP with M_w_ = 360 kDa
H40	60	4	40	0.21	AIBN	0	PVP with M_w_ = 40 kDa
HACVA	60	4	1300	0.21	ACVA	0	ACVA as initiator
HMA	60	4	1300	0.21	AIBN	9:1	Addition of MA

**Table 2:** Summary of the EWC, water uptake, and the degree of conversion after 24 h, all expressed in %, for the synthesized gels.

Gel	EWC (%)	Water Uptake (%)	Degree of Conversion (%)
H65	215	103	72
H70C	167	82	94
H16h	152	58	60
HMBA2	92	51	53
HMBA3	70	48	50
HMBA5	49	44	48
H360	200	79	86
H40	n.d.	n.d.	n.d.
HACVA	160	25	96
HMA	170	85	84

n.d. = not detected.

**Table 3:** Summary of the T_g_ values (°C) that were obtained from DSC thermograms for the synthesized gels and the constituent polymeric species.

Sample	T_g_ (°C)
PVP (M_W_ = 1300 kDa)	181
P(HEMA)	124
H65	168
H360	169
HMA	167

**Table 4:** Parameters of the EPR signals that were observed on different gels after 60 min contact with laboratory-stained marble specimens.

Gel	g_1_ ± 0.005	g_2_ ± 0.005	g_3_ ± 0.005	A_1_ ± 5 (G)	A_2_ ± 5 (G)	A_3_ (G)
H360	2.245	2.071	1.999	187	13	<3
HMA	2.252	2.071	2.000	182	12	<3
H65	2.258	2.072	2.006	176	12	<3

**Table 5:** Chromatic parameters (L*, a*, b*) of marble specimens before (t_1_) and after (t_2_) contact with H65 and HMA gels for 60 min.

	t_1_			t_2_			
Contacted Gels	L*	a*	b*	L*	a*	b*	∆E*
H65	81.04 ± 0.84	−10.34 ± 0.61	5.97 ± 0.74	85.81 ± 1.52	−6.52 ± 0.66	6.19 ± 0.75	6.15
HMA	86.33 ± 0.65	−8.10 ± 0.84	2.93 ± 0.56	87.26 ± 0.81	−2.44 ± 0.24	2.61 ± 0.54	5.74

## Data Availability

Not applicable.

## References

[1] Xu B. (2009). Gels as Functional Nanomaterials for Biology and Medicine. Langmuir.

[2] Kopečhek J. (2009). Hydrogels: From Soft Contact Lenses and Implants to Self-Assembled Nanomaterials. J. Polym. Sci. Part A Polym. Chem..

[3] Fratini E., Carretti E., Baglioni P., Chelazzi D. (2013). Cleaning IV: Gels and Polymeric Dispersions. Nanoscience for the Conservation of Works of Art.

[4] Ullah F., Othman M.B.H., Javed F., Ahmad Z., Akil H.M. (2015). Classification, processing and application of hydrogels: A review. Mater. Sci. Eng. C.

[5] Sansonetti A., Bertasa M., Canevali C., Rabbolini A., Anzani M., Scalarone D. (2020). A review in using agar gels for cleaning art surfaces. J. Cult. Herit..

[6] Bertasa M., Dodero A., Alloisio M., Vicini S., Riedo C., Sansonetti A., Scalarone D., Castellano M. (2020). Agar gel strength: A correlation study between chemical composition and rheological properties. Eur. Polym. J..

[7] Sansonetti A., Bertasa M., Corti C., Rampazzi L., Monticelli D., Scalarone D., Sassella A., Canevali C. (2021). Optimization of Copper Stain Removal from Marble through the formation of Cu(II) Complexes in Agar Gels. Special Issue Gels Horizon: From Science to Smart Materials. Gels.

[8] Sansonetti A., Casati M., Striova J., Canevali C., Anzani M., Rabbolini A. A cleaning method based on the use of agar gels: New tests and perspectives. Proceedings of the 12th International Congress on the Deterioration and Conservation of Stone.

[9] Bertasa M., Bandini F., Felici A., Lanfranchi M.R., Negrotti R., Riminesi C., Scalarone D., Sansonetti A. (2018). Soluble salts extraction with different thickeners: Monitoring of the effects on plaster. IOP Conf. Ser. Mater. Sci. Eng..

[10] Canevali C., Fasoli M., Botteon A., Bertasa M., Colombo A., Di Tullio V., Capitani D., Proietti N., Scalarone D., Sansonetti A. (2016). A multi-analytical approach for the study of copper stain removal by agar gels. Microchem. J..

[11] Bertasa M., Canevali C., Sansonetti A., Lazzari M., Malandrino M., Simonutti R., Scalarone D. (2021). An in-depth study on the agar gel effectiveness for built heritage cleaning. J. Cult. Herit..

[12] Bertasa M., Chiantore O., Poli T., Riedo C., Di Tullio V., Canevali C., Sansonetti A., Scalarone D. (2017). A Study of Commercial Agar Gels as Cleaning Materials. Proceedings of the Gels in Conservation.

[13] Angelova L.V., Ormsby B., Townsend J.H., Wolbers R. (2017). Gels in the Conservation of Art.

[14] Campani E., Casoli A., Cremonesi P., Saccani I., Signorini E. (2007). Use of Agarose and Agar for preparing “Rigid Gels”. Quaderni del Cesmar.

[15] Pizzorusso G., Fratini E., Eiblmeier J., Giorgi R., Chelazzi D., Chevalier A., Baglioni P. (2012). Physico-chemical characterization of acrylamide/bisacrylamide hydrogels and their application for the conservation of easel paintings. Langmuir.

[16] Alemàn J., Chadwick A.V., He J., Hess M., Horie K., Jones R.G., Kratochvìl P., Meisel I., Mita I., Moad G. (2007). Definitions of terms relating to the structure and processing of sols, gels, networks, and inorganic-organic hybrid materials (IUPAC Recommendations 2007). Pure Appl. Chem..

[17] Domingues J.A.L., Bonelli N., Giorgi R., Baglioni P. (2014). Chemical semi-IPN hydrogels for the removal of adhesives from canvas paintings. Appl. Phys. A.

[18] Domingues J.A.L., Bonelli N., Giorgi R., Fratini E., Baglioni P. (2013). Innovative method for the cleaning of water-sensitive artifacts: Synthesis and application of highly retentive chemical hydrogels. Int. J. Conserv. Sci..

[19] Baglioni P., Chelazzi D., Giorgi R. (2015). Nanotechnologies in the Conservation of Cultural Heritage.

[20] Nkansah P., Antipas A., Lu Y., Varma M., Rotter C., Rago B., El-Kattan A., Taylor G., Rubio M., Litchfield J. (2013). Development and evaluation of novel solid nanodispersion system for oral delivery of poorly water-soluble drugs. J. Control. Release.

[21] Skorokhoda V., Semenyuk N., Melnyk J., Suberlyak O. (2009). Hydrogels penetration and sorption properties in the substances release controlled processes. Chem. Chem. Technol..

[22] Suberlyak O., Melnyk J., Skorokhoda V. (2009). Formation and properties of hydrogel membranes based on cross-linked copolymers of methacrylates and water-soluble polymers. Eng. Biomater..

[23] Skorokhoda V., Melnyk Y., Semenyuk N., Ortynska N., Suberlyak O. (2017). Film Hydrogels on the basis of polyvinylpyrrolidone copolymers with regulated sorption-desorption characteristics. Chem. Chem. Technol..

[24] Melnyk Y., Stetsyshyn Y., Skorokhoda V., Nastishin Y. (2020). Polyvinylpyrrolidone-graft-poly(2-hydroxyethylmethacrylate) hydrogel membranes for encapsulated forms of drugs. J. Polym. Res..

[25] Domingues J.A.L., Bonelli N., Giorgi R., Fratini E., Gorel F., Baglioni P. (2013). Innovative hydrogels based on semi-interpenetrating p(HEMA)/PVP networks for the cleaning of water-sensitive cultural heritage artifacts. Langmuir.

[26] Hasanzadeh R., Mogdaham P.N., Samadi N. (2013). Synthesis and application of modified poly(styrene-alt-maleic anhydride) networks as a nano resin for uptake of heavy metals. Polym. Advan. Technol..

[27] McMullen R.L., Musa O.M. (2016). Maleic anhydride applications in personal care. Handbook of Maleic Anhydride based Materials.

[28] Hood D.K., Musa O.M., Musa O.M. (2016). Application of maleic anhydride in coatings, adhesives and printing. Handbook of Maleic Anhydride based Materials.

[29] Kumar R., Al-Haddad S., Al-Rughaib M., Salman M. (2016). Evaluation of hydrolyzed poly(isobutylene-alt-maleic anhydride) as a polyelectrolyte draw solution for forward osmosis desalination. Desalination.

[30] Liu Q., Hedberg E.L., Liu Z., Bahulekar R., Meszlenyi R.K., Mikos A.G. (2000). Preparation of macroporous poly(2-hydroxyethylmethacrylate) hydrogels by enhanced phase separation. Biomaterials.

[31] Monticelli D., Castelletti A., Civati D., Recchia S., Dossi C. (2019). How to efficiently produce ultrapure acids. Int. J. Anal. Chem..

[32] Stiles W. (2000). Color Science: Concepts and Methods, Quantitative Data and Formulae.

[33] Anania L., Badalà A., Barone G., Belfiore C.M., Calabrò C., La Russa M.F., Mazzoleni P., Pezzino A. (2012). The stones in monumental masonry buildings of the “Val di Noto” area: New data on the relationships between petrographic characters and physical-mechanical properties. Constr. Build. Mater..

[34] Peisach J., Blumberg W. (1974). Structural implications derived from the analysis of electron paramagnetic resonance spectra of natural and artificial copper proteins. Arch. Biochem. Biophys..

[35] Canevali C., Morazzoni F., Scotti R., Cauzzi D., Moggi P., Predieri G. (1999). Electron paramagnetic resonance characterisation of silica-dispersed copper molybdate obtained by sol-gel and impregnation methods. J. Mater. Chem..

[36] Zhang N., Zhou Q., Yin X., Zeng D. (2014). Trace Amounts of Aqueous Copper(II) Chloride Complexes in Hypersaline Solutions: Spectrophotometric and Thermodynamic Studies. J. Solut. Chem..

